# Global Dissemination of a Single Mutation Conferring White Pericarp in Rice

**DOI:** 10.1371/journal.pgen.0030133

**Published:** 2007-08-10

**Authors:** Megan T Sweeney, Michael J Thomson, Yong Gu Cho, Yong Jin Park, Scott H Williamson, Carlos D Bustamante, Susan R McCouch

**Affiliations:** 1 Department of Plant Breeding and Genetics, Cornell University, Ithaca, New York, United States of America; 2 International Rice Research Institute, Los Baños, Philippines; 3 Indonesian Center for Agricultural Biotechnology and Genetic Resources Research and Development, Bogor, Indonesia; 4 Department of Agronomy, Chungbuk National University, Chongju, Republic of Korea; 5 National Institute of Agricultural Biotechnology, Suwon, Republic of Korea; 6 Department of Biological Statistics and Computational Biology, Cornell University, Ithaca, New York, United States of America; University of Chicago, United States of America

## Abstract

Here we report that the change from the red seeds of wild rice to the white seeds of cultivated rice *(Oryza sativa)* resulted from the strong selective sweep of a single mutation, a frame-shift deletion within the *Rc* gene that is found in 97.9% of white rice varieties today. A second mutation, also within *Rc,* is present in less than 3% of white accessions surveyed. Haplotype analysis revealed that the predominant mutation originated in the *japonica* subspecies and crossed both geographic and sterility barriers to move into the *indica* subspecies. A little less than one Mb of *japonica* DNA hitchhiked with the *rc* allele into most *indica* varieties, suggesting that other linked domestication alleles may have been transferred from *japonica* to *indica* along with white pericarp color. Our finding provides evidence of active cultural exchange among ancient farmers over the course of rice domestication coupled with very strong, positive selection for a single white allele in both subspecies of O. sativa.

## Author Summary

Understanding the history and origin of genetic mutations that have changed wild plants into crops can help us understand the history of the people who cultivated these plants. Rice is one of the oldest crops grown in Asia and it contains two different subspecies that are believed to have been domesticated in different locations by different people. Surprisingly, some of the genetic mutations responsible for domestication are common in all rice. We here show that a mutation in the *Rc* gene that changed the red seed of wild rice into the white seeds of modern rice is shared by a large majority of all rice varieties, regardless of subspecies. This transfer of genes requires contact among rice types and implies contact among the people who cultivated the different subspecies. We have traced the origin of the mutation in *Rc* to the *japonica* subspecies. As additional domestication genes are cloned and their evolutionary history described, we will see how many times and in how many directions such gene transfers have occurred.

## Introduction

Human efforts to domesticate plant and animal species began thousands of years before recorded history, leaving us to guess at the methods that transformed wild species into agriculturally important crops and livestock. Rice, *Oryza sativa,* was domesticated in Asia and is now grown on every continent throughout the world, with the exception of Antarctica. The species consists of two major subspecies, *indica* and *japonica,* whose separate genetic identities were recognized in ancient times and are maintained by sterility barriers coupled with the inbreeding habit of O. sativa [1–3]*.* The *indica* and *japonica* subspecies can be further subdivided into five genetically distinct subpopulations. Several estimates have been made for the time of divergence between *indica* and *japonica* based on intron sequence and retrotransposon insertions, and all of these calculations place the time to the recent common ancestor at more than 100,000 years ago [4–6]. This divergence time is an order of magnitude larger than the oldest estimates for rice domestication. The data strongly support at least two independent domestications of O. sativa from predifferentiated pools of the O. rufipogon wild ancestor [3,4,7,8].

With such evidence for independent domestications, we would expect to see different mutations conditioning domestication traits fixed within the different subspecies. We recently cloned the *Rc* gene, a bHLH protein, which is the only reported locus in rice that effects a change from red grain to white grain [9]. The change in pericarp color from red to white is an important hallmark of rice domestication. Most rice cultivars grown and consumed throughout the world today have a white or beige pericarp, the color of unpolished “brown rice” while all known accessions of the wild ancestor, *O. rufipogon,* invariably have grains with a red pericarp. Here, we investigate the number and origin of mutations that give rise to diverse white-grained rice cultivars from around the world, and the extent to which they were disseminated throughout the genetically and geographically diverse range of *O. sativa.* The evolutionary history of the *Rc* locus provides clear documentation that a single mutation moved rapidly from the *japonica* into the *indica* subspecies, while an independent mutation in the *aus* subpopulation was not widely disseminated during rice domestication.

## Results

### How Many Independent Mutations Are Responsible for White Pericarp in O. sativa?

**Figure 1:**
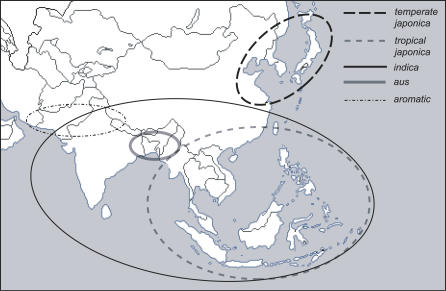
Current Distribution of the Five Major Subpopulations of Rice in Asia For details about the geographical distribution of accessions used in this study, see Table S1.

To determine the frequency and distribution of the 14-bp deletion in the *Rc* gene that has been shown to cause white pericarp in rice [9,10], a set of 440 geographically and genetically diverse rice cultivars were genotyped using the rice indel RID 12 primers reported in Sweeney et al. (2006). Our panel of varieties was selected to represent the range of diversity within Asian cultivated rice*.* It included both landrace and modern varieties from the five well-defined subpopulations of O. sativa [3] (Figure 1) (204 *indica,* 33 *aus,* 87 *temperate japonica,* 99 *tropical japonica,* and 17 *aromatic* rices) collected in 24 different countries on three different continents (Table S1). Molecular polymorphism data groups the *indica* and *aus* subpopulations within the *indica* subspecies and the *tropical japonica, temperate japonica,* and *aromatic* subpopulations within the *japonica* subspecies [2,3].

**Figure 2:**
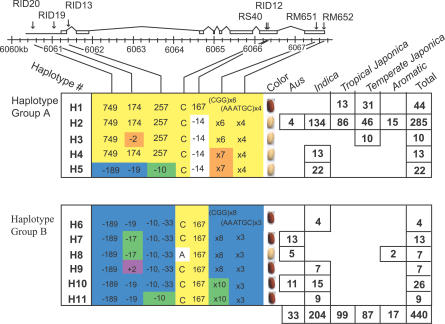
Haplotype Data across the *Rc* Gene The gene model for *Rc,* containing seven exons and comprising a region of approximately 8 kb, is shown horizontally along the top; arrows on the gene model and promoter region of *Rc* indicate positions of markers: RID-number markers amplify rice insertion/deletion polymorhisms, RM-number markers amplify rice microsatellite polymorphisms, and the RS-number marker detects a rice SNP. Physical positions along the rice Chromosome 7 pseudomolecule are indicated by the ruler below the gene model. In the tables below, allele sizes for RID marker loci are given in bp and allele sizes for RM markers show the number of repeats at each microsatellite locus. The number of varieties in each subpopulation that carry each haplotype is provided in boxed squares in the table to the right. Haplotypes found in varieties with red pericarp are shaded red.

One hundred percent of the varieties from the *indica, tropical japonica,* and *temperate japonica* populations (*n* = 311) with white pericarp contained an identical 14-bp deletion in the bHLH gene; this deletion was found in none of those with red pericarp (*n* = 103) (Figure 2). This mutation was also found in 15 of the 17 white *aromatic* and four of the nine white *aus* varieties. Thus, a single 14-bp deletion in the *Rc* gene is present in both subspecies and in all five subpopulations and is responsible for white pericarp in 97.9% (330/337) of rice varieties surveyed.

To identify mutations that could explain the lack of pigment in the seven white rice varieties that did not contain the 14-bp deletion, we sequenced the entire coding region of *Rc* in two of the white *aus* varieties and one white *aromatic* variety that lacked the 14-bp deletion. As a control, nine red pericarp varieties from different subpopulations were also sequenced. A SNP (C→A) in exon 6 of the *Rc* gene distinguished these white- and red-grained rices. This SNP introduces a premature stop codon that truncates the protein before the bHLH domain. Further sequencing confirmed that this SNP was predictive of white pericarp in all seven white rices that did not contain the 14-bp deletion in *Rc* (DQ902346-DQ902352). Thus, the C→A SNP in exon 6 represents an independent mutation in the *Rc* gene that was not found in any of the *indica, tropical,* or *temperate japonica* varieties but exists at moderate frequency in white *aus* varieties (five of nine, 55.5%) and in two accessions out of 17 *aromatic* cultivars (Figure 2).

### Ancestral Haplotypes and the Origin of *Rc* Mutations

To determine the subpopulation origin of the *Rc* mutations, we examined ancestral haplotypes across the *Rc* coding sequence and promoter region in 103 genetically diverse, red-grained rices. Four rice insertion/deletion polymorphism (RID) and two rice microsatellite (RM) polymorphisms were used to construct haplotypes across the 6.5-kb region containing the *Rc* gene (Figure 2). A single haplotype, H1, was detected among the 44 red-grained *tropical* and *temperate japonica* plants; we will refer to this as the ancestral *japonica* haplotype. No red rices belonging to the *aromatic* subpopulation were available for in this study. Five closely related haplotypes were identified among the 24 red *aus* and 35 red *indica* landraces, and all are clearly differentiated from the *japonica* haplotype. Haplotypes 6–7 and 9–11 (Figure 2) are derivatives of each other and the similarity among them reflects the close evolutionary relationship between the *indica* and *aus* subpopulations [3]. When all red-grained *indica, aus,* and *japonica* accessions (*n* = 103) are compared, it can be seen that they share the wild-type alleles at both functional markers (the non-deletion allele at RID12 and the C allele at SNP marker RS40, Figure 2). All flanking marker alleles were unique to one of the subspecies or subpopulation groups. Additionally, we collected ∼6,350 bp of sequence data for the *Rc* gene and ∼650 bp of downstream DNA from four red *indicas* and four red *japonica* varieties. Within this 7-kb region there are 21 SNPs whose alleles are subspecies specific. Thus, there are clearly differentiated haplotypes that distinguish the ancestral *japonica* gene pool (Haplotype Group A) from the ancestral *indica* and *aus* gene pools (Haplotype Group B) across this genic region. Based on these ancestral haplotypes, we were able to trace the origin of both functional mutations leading to white pericarp.

The most common haplotype of white pericarp rices, H2, contained the 14-bp deletion at RID12, and differed from the ancestral *japonica* haplotype, H1, only at this functional mutation. In contrast, the white H2 haplotype differed from the *aus* and *indica* red haplotypes (Haplotype Group B) at every marker tested (Figure 2). This provides strong support for the conclusion that the H2 haplotype originated in a *japonica* ancestor. White haplotypes H3 and H4 are derived from H1, differing at a single marker locus in each case. Haplotype 5 provides evidence of a putative recombination event. It is found only in *indica* accessions and cultivars carrying this haplotype have ancestral *indica* alleles across the promoter and first intron of *Rc* and ancestral *japonica* alleles across the remaining sequence of the gene*,* including the 14-bp deletion. These data strongly support a single origin of the 14-bp deletion from a *japonica* background that then was introgressed into *indica* and *aus*.

**Figure 3:**
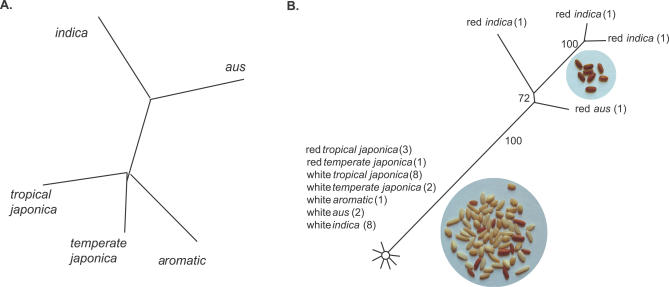
Comparison of *Rc* Gene and Rice Genome-Wide Phylogenetic Trees Sequence alignment adjusted manually in MacClade 4.05. A heuristic parsimony search was conducted on the data matrix, excluding gapped characters, with TBR branch swapping. This identified a single tree at 53 steps, with a consistency index of 0.98 (0.97 without autapomorphies) and a retention index of 0.99. Bootstrap support was estimated using 100 replications of the same search strategy. Pericarp color and subpopulation of each variety sequenced is labeled.

Sequence data (7 kb across *Rc,* Table S2) from white accessions with the deletion (eight *indica,* two *aus,* eight *tropical japonica,* two *temperate japonica,* and one *aromatic*) and eight red accessions from both the *indica* and *japonica* subspecies were used to create a gene tree (Figure 3). If the *Rc* gene tree and the tree created using genome-wide SSR data [3] had similar typologies, it would suggest an independent origin of white pericarp in *indica* and *japonica*. However, these trees are not consistent, suggesting introgression from one subspecies into the other. Accessions with red pericarp share a similar distribution pattern in both tress, but all whites with the deletion have *japonica* alleles at the 21 subspecies specific SNPs within the *Rc* gene and form a cluster with the red *japonicas* regardless of their subpopulation identity (Figure 3). Thus, phylogenetic analysis confirms the *japonica* origin of the 14-bp deletion. Together, the lack of concordance between the *R*c gene and rice genomic trees and the marker analysis provides strong support for the conclusion that the 14-bp deletion conferring white pericarp in rice arose once in the *japonica* gene pool and was widely introgressed into *indica* and *aus* landraces*.* The presence of this deletion within 97.9% of white-grained rice varieties found throughout the world today suggests either that the gene was dispersed during the early phases of domestication and is common by descent in modern varieties or a that very strong, positive selection for the allele lead to its introgression and maintenance in already established gene pools.

The second mutation *Rc-s* is only found in haplotype 8 and is restricted to six *aus* and two *aromatic* accessions. These white-grained varieties share no marker alleles with any of the *tropical* or *temperate japonica* accessions, suggesting that the origin of this allele is different from that of the 14-bp deletion. H8 differs from H7, a red *aus* haplotype, by only the functional C→A SNP. Since H7 is restricted to the *aus* subpopulation, we conclude that the C→A functional polymorphism originated in the *aus* subpopulation and that this allele was not widely disseminated during the domestication of O. sativa.

### How Extensive Was Genetic Hitchhiking around the *rc* Allele?

**Figure 4:**
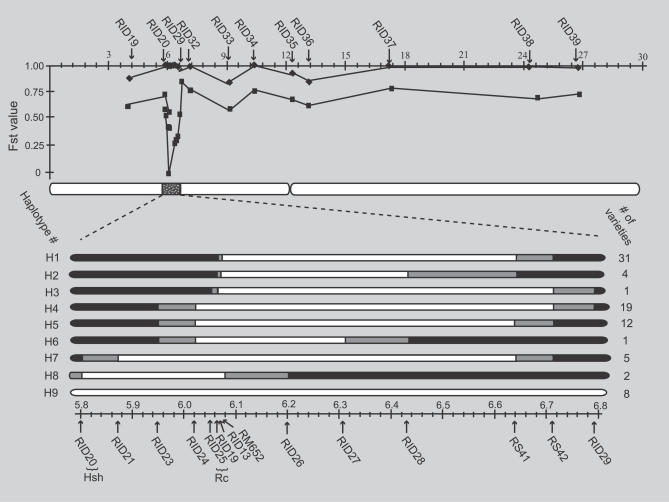
*Japonica* Introgressions in White *Indica* Arrows indicate the location of markers on the physical map of Chromosome 7, numbers are in Mb. *F*_ST_ values for red *indica* versus *japonica* are plotted across the chromosome using diamonds, white *indica* versus *japonica* are plotted using squares. The expanded region shows a detailed view of the 1-Mb window around the *Rc* gene. All varieties genotyped are white *indicas* containing the 14-bp deletion. DNA segments containing alleles of *japonica* ancestry (in the *indica* background) are shown in white, segments containing alleles of *indica* ancestry are shown in black, and segments containing a breakpoint between alleles of *japonica* and *indica* ancestry are shown in grey. Locations of the *Rc* and *Hsh* genes indicated by brackets.

How much DNA of *japonica* ancestry was introgressed into *indica* cultivars along with the 14-bp deletion conferring white pericarp is of particular interest because the *Rc* locus falls within the map positions of several quantitative trait loci associated with other domestication-related traits (dormancy, shattering, tillering, and panicle architecture) on Chromosome 7 [11–17]. It is possible that an array of domestication alleles arose in the *japonica* background and were transferred as a block into *indica* cultivars. In order to define the extent of the introgression, we designed a series of indel and SNP markers that clearly distinguished the ancestral *indica* and *japonica* gene pools across Chromosome 7. To identify these markers, we evaluated polymorphism on a panel of 30 *japonicas* and 15 red-seeded *indicas.* Red *indicas* contain ancestral *indica* sequence in this region while both red and white *japonicas* (as well as white *indicas*) are expected to contain ancestral *japonica* sequence. *F*_ST_ values provide a quantitative estimate of the degree of allelic differentiation between subpopulations. The indel and SNP markers used to distinguish the ancestral gene pools all had *F*_ST_ values above 0.8 (see upper portion of Figure 4).

These markers were used to genotype 88 diverse white *indica* varieties to explore the extent of *japonica* DNA in the *Rc* region (Table S3). In this study, nine extended haplotype patterns were identified and they showed varying sizes of *japonica* introgressions (Figure 4, upper portion). Ninety-one percent of the *indica* varieties contained less than 1 Mb of *japonica* derived DNA in the region, with asymmetric distribution around *rc.* This is similar to haplotype patterns described for the *Arabidopsis FRI* gene [18] The haplotype with the smallest introgression, H2, which was observed in two varieties, was only 247–371 kb in size (Figure 4, lower portion) and contained approximately 100 genes. Ten white *indica* varieties, H8 and H9, contained an introgression that included the recently mapped shattering locus, *Hsh* [14], although in most white *indicas* the *japonica* introgression does not extend that far. In an extreme case, H9, eight *indica* landraces collected from an isolated population in Kalimantan, Indonesia (on the island of Borneo) had *japonica*-derived alleles across the entire length of Chromosome 7 (29.7 Mb) (Figure 4).

When *F*_ST_ values were calculated using data from 88 white *indicas* and 30 *japonicas*, we observed a dramatic drop around *Rc,* from values of over 0.75, down to 0 and back above 0.75 within a 1-Mb region (Figure 4, upper portion). *F*_ST_ values around 0 indicate no differentiation between subpopulations and the drop around *Rc* illustrates the location and size of the *japonica* introgression (Figure 4). The difference in *F*_ST_ values outside the *Rc* region between the red or white *indicas* compared to *japonica* is due to the inclusion of the eight white *indicas* from Kalimantan that carry *japonica* alleles across the entire length of Chromosome 7. As more domestication genes are cloned from this region, the pattern and extent of introgression reported here can be used to determine if *japonica* alleles for other domestication traits hitchhiked along with the *rc* mutation when it was introduced into *indica* landrace varieties.

### Genetic Diversity in *Rc*

To investigate the reduction in diversity around the 14-bp deletion in *Rc,* we used sequences from a portion of the *Rc* gene in 21 diverse varieties of white rice carrying the 14-bp deletion and eight red varieties representing *indica*, *aus*, *tropical,* and *temperate japonica* subpopulations (Table S2). A summary of DNA polymorphism in the *Rc* gene is given in Table 1. Levels of polymorphism are reduced by 98% in the white pericarp rices, compared to the red landraces. Notably, the levels of DNA polymorphism in red landraces is comparable to randomly chosen loci in an unbiased sample of O. sativa landraces. For red landraces in the *Rc* region π (the level of nucleotide diversity) = 0.0025 per base pair, whereas in a survey of randomly chosen loci in *O. sativa,* π = 0.0032 (Ana Caicedo and Scott Williamson, personal communication, University of Massachusetts, Amherst, Massachussetts and Cornell University, respectively). Thus, other than the extreme reduction in diversity in white pericarp rices, the *Rc* region is not atypical of the rice genome. Furthermore, the site-frequency spectrum of the white rice sample is completely skewed towards polymorphisms with rare derived alleles. All of these observations are consistent with very strong, recent selection in favor of the 14-bp deletion in the *Rc* gene.

**Table 1:**
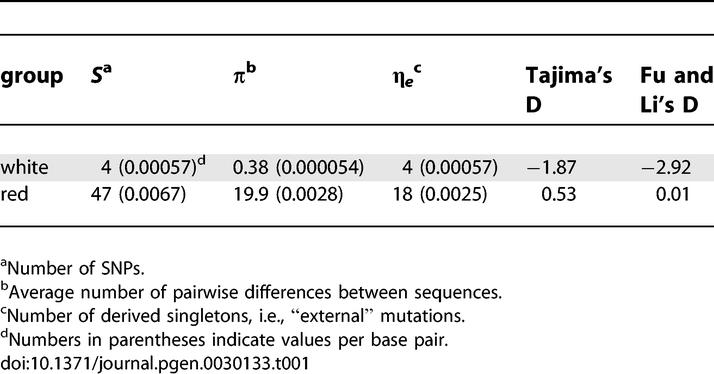
DNA Sequence Polymorphism in the *Rc* Gene

## Discussion

Here, we show that the previously described 14-bp deletion in *Rc* leading to white pericarp in rice is found in over 97% of white pericarp rice varieties surveyed, including landrace representatives from all of the major rice subpopulations. This allele arose in a *japonica* background and was introgressed into the other subpopulations. We determined that the size of the *japonica* introgression surrounding *rc* in many *indica* varieties is less than 1 Mb.

A second mutation leading to white pericarp in 3% of rice varieties surveyed was identified and shown to have arisen in an *aus* background. This mutation is identical to the one described by [9] for the *Rc-s* allele conditioning light red or amber pericarp in the *aus* variety Surjamkuhi. As previously reported, the C→A transversion in exon 6 of the *Rc* gene results in a premature stop codon that truncates the protein before the bHLH domain. While it is not surprising that a truncated version of the bHLH protein would result in white pericarp, it is curious that the same truncated protein leads to light red pericarp in some genetic backgrounds. The explanation is expected to lie in the fact that bHLH proteins are found in complexes in maize, *Arabidopsis,* and petunia [19–21], and different alleles of the interacting proteins are likely to determine the phenotype when *Rc* is truncated by the C→A substitution. A segregating population derived from a cross between white and light red varieties, both carrying the *Rc-s* allele, could pinpoint the genetic cause(s) of the difference in phenotype.

The lack of global dissemination of the *Rc-s* allele leading to either white or light-red pericarp may be partly a question of timing. If the *Rc-s* allele occurred after the *rc* allele (14-bp deletion) had become prevalent, there would have been no strong selective pressure driving its spread. The fact that in some genetic backgrounds the *Rc-s* allele produces a light red grain may be another reason why this allele was considered less desirable for early farmers, reducing the selective advantages and limiting its distribution.

### How Was the *rc* Allele Disseminated across Asia?

In an outcrossing species, it is not surprising to see the rapid spread of a favorable mutation. However, O. sativa is 97%–98% inbreeding [1,22], has a closed-flower morphology, and short-lived pollen grains. Gene flow is further constrained by the presence of a complex network of sterility barriers between the two independently domesticated *indica* and *japonica* subspecies of rice [1,23]. Even among largely out-crossing wild *Oryza* relatives, viable pollen rarely travels more than 10 m [24], making it difficult for a favorable allele to be widely disseminated through natural pollen flow. Thus, it is unlikely that the *rc* mutation would have traveled far beyond its point of origin if not for the activities of humans who valued the white grains as a source of food and presumably as a commodity for trade. The importance of the *rc* mutation to early agriculturalists is evidenced by the fact that it moved around the Himalayan mountain range that is found between the proposed centers of *indica* and *japonica* domestication [4,8], and, having traversed this substantial geographic barrier, was rapidly introgressed into all major subpopulations of rice despite an emerging fertility barrier.

Three key genetic features of white pericarp contributed to the rapid spread of this trait. First, *rc* is a single gene mutation that causes a qualitative change in phenotype so it is straightforward to visually distinguish red from white grains. However, the color of the seed coat is not visible on plants in the field because each grain of rice is completely covered by a hull, or glume. As dehulling reduces the germination rate, seeds that were to be planted the following season would be maintained with the hulls on. The pericarp color would only have been obvious to those who dehulled the grains, something that is normally done just prior to cooking. The pericarp is a maternally derived cell layer and its color is determined by the maternal genotype rather than the genotype of the fertilized embryo. Therefore, all seeds on any one plant are the same color. By dehulling only a few grains on any plant, it would be apparent which plants carried red or white seeds, allowing for selection. Third, the *rc* mutation is recessive, and in a highly self-pollinated species such as O. sativa, this means that the trait breeds true; seeds with white pericarp will produce offspring having grains with white pericarp for generations to come.

### Why Did Farmers Prefer White Rice?

Almost all wild plants have pigmented seed coats, skin, and flesh, yet humans seem to prefer white starchy staples, and selection against pigmentation is a common theme in the evolutionary history of crop plants. The reasons our ancestors selected against pigmentation in these staple foods are not entirely clear, although it is tempting to speculate.

The novelty of a different color rice may have contributed to its popularity. White rice has the very practical advantage that it is far easier to detect and eliminate insects and pathogens against a light background than a darker red background. Alternatively, differences in cooking properties between red and white rice may have resulted in the preference for white. Colored rice has a harder seed coat than white rice. This means longer cooking times as well as more time spent finding fuel with which to cook. In many cultures, the rice hull and bran layers were removed by pounding. As red rice has a harder seed coat than white, it requires more work to remove the bran layer from reds. No matter what the reason for selecting against red pericarp in rice, selection was strong enough to catalyze a major change from red to white grains across a vast geographic area in a surprisingly short period of time.

Despite the lack of human records, the history of domestication is written in the genomes of the plants and animals that have been changed by our choices. As we begin to decipher these stories, we gain new insight into our own history as part of the domestication process. In rice, at least two important and unlinked domestication alleles, *rc* (discussed in this paper) and the shattering allele, *sh4* (located on Chromosome 4) [25], are now known to have arisen only once in evolution and to have been introgressed across the *indica–japonica* divide, with almost complete prevalence in modern forms of cultivated rice. Other loci affecting domestication traits, such as the *Rc-s* allele for white pericarp (this paper) or the *sh1* allele for non-shattering [26], confer alternative or complementary alleles for the same domestication phenotypes, but their distribution is restricted to a specific subpopulation. The fact that cultivars belonging to the deeply divergent *indica* and *japonica* subspecies have both common and distinct domestication genes suggests that the process of domestication in O. sativa involves complex patterns of subpopulation isolation and convergence, underwritten by a rich tapestry of cross-talk among ancient farmers. Whether a majority of key domestication alleles are subpopulation-specific or are shared among all Asian rice varieties, and whether the shared alleles arose predominantly within a single subspecies or subpopulation, is not yet known. The answer to this question will illuminate the paths traveled by ancient peoples and their innovations in the rice-growing world.

## Materials and Methods

### PCR and primer development.

List of primers in Table S4 All primers have an annealing temperature of 55 °C. For the introgression study, primers were designed to amplify small (4–30 bp) insertion/deletion events that were originally detected computationally by aligning the sequences from cv Nipponbare *(japonica)* and cv 9311 *(indica)* [27,28]. As 9311 is a white *indica*, it carries a *japonica* introgression around *rc*. In order to identify indels between ancestral *indica* and *japonicas* in this region, short stretches of sequence from the red *indica* cv Mudgo were aligned with Nipponbare. To determine the pericarp color, ten seeds per plant were dehulled and then visually inspected.

### Phylogenetic tree construction.

*Rc* DNA sequenced using overlapping primers. SNPs were confirmed with multiple reads through independent amplicons. Amplified products were sequenced at the Cornell Biotechnology Resource Center. Alignment adjusted manually in MacClade 4.05 (http://macclade.org/macclade.html). A heuristic parsimony search was conducted on the data matrix, excluding gapped characters, with TBR branch swapping. This identified a single tree at 53 steps, with a consistency index of 0.98 (0.97 without autapomorphies) and a retention index of 0.99. Bootstrap support was estimated using 100 replications of the same search strategy.

## Supporting Information

Table S1 List of Rice Varieties and Corresponding Genotypes for the Association Study (123 KB XLS)

Table S2 Sequence Polymorphisms across the *Rc* Gene for 29 Rice Varieties (23 KB XLS)

Table S3 List of Rice Varieties and Corresponding Genotypes Used for the Introgression Study (61 KB XLS)

Table S4 Primers Used in This Study (17 KB XLS)

### Accession Numbers

The sequences discussed in this paper were assigned National Center for Biotechnology Information (NCBI) GenBank (http://www.ncbi.nlm.nih.gov/gquery/gquery.fcgi) accession numbers DQ885795–DQ885823 and DQ902346–DQ902352.

## Abbreviations

RID: rice insertion/deletion polymorphism
RM: rice microsatellite

## References

[pgen-0030133-b001] Oka HI (1988). *Indica-Japonica* differentiation of rice cultivars. Origin of cultivated rice.

[pgen-0030133-b002] Glaszmann JC (1987). Isozymes and classification of Asian rice varieties. Theor Appl Genet.

[pgen-0030133-b003] Garris AJ, Tai TH, Coburn J, Kresovich S, McCouch S (2005). Genetic structure and diversity in Oryza sativa L. Genetics.

[pgen-0030133-b004] Vitte C, Ishii T, Lamy F, Brar D, Panaud O (2004). Genomic paleontology provides evidence for two distinct origins of Asian rice (Oryza sativa L.). Mol Genet Genomics.

[pgen-0030133-b005] Vaughan DA, Morishima H, Kadowaki K (2003). Diversity in the *Oryza* genus. Curr Opin Plant Mol Biol.

[pgen-0030133-b006] Ma J, Bennetzen JL (2004). Rapid recent growth and divergence of rice nuclear genomes. Proc Natl Acad Sci U S A.

[pgen-0030133-b007] Second G (1982). Origin of the genic diversity of cultivated rice (Oryza spp.): Study of the polymorphism scored at 40 isozyme loci. Jpn J Genet.

[pgen-0030133-b008] Londo JP, Chiang Y, Hung K, Chiang T, Schaal BA (2006). Phylogeography of Asian wild rice, *Oryza rufipogon,* reveals multiple independent domestications of cultivated rice, Oryza sativa. Proc Natl Acad Sci U S A.

[pgen-0030133-b009] Sweeney MT, Thomson MJ, Pfeil BE, McCouch S (2006). Caught red-handed: Rc encodes a basic helix-loop-helix protein conditioning red pericarp in rice. Plant Cell.

[pgen-0030133-b010] Furukawa T, Maekawa M, Oki T, Suda I, Iida S (2007). The *Rc* and *Rd* genes are involved in proanthocyanidin synthesis in rice pericarp. Plant J.

[pgen-0030133-b011] Gu XY, Kianian SF, Foley ME (2005). Phenotypic selection for dormancy introduced a set of adaptive haplotypes from weedy into cultivated rice. Genetics.

[pgen-0030133-b012] Gu XY, Kianian SF, Hareland GA, Hoffer BL, Foley ME (2005). Genetic analysis of adaptive syndromes interrelated with seed dormancy in weedy rice *(Oryza sativa)*. Theor Appl Genet.

[pgen-0030133-b013] Gu XY, Kianian SF, Foley ME (2004). Multiple loci and epistases control genetic variation for seed dormancy in weedy rice *(Oryza sativa)*. Genetics.

[pgen-0030133-b014] Ji HS, Chu SH, Jiang W, Cho YI, Hahn JH (2006). Characterization and mapping of a shattering mutant in rice that corresponds to a block of domestication genes. Genetics.

[pgen-0030133-b015] Li C, Zhou A, Sang T (2006). Genetic analysis of rice domestication syndrome with the wild annual species, Oryza nivara. New Phytol.

[pgen-0030133-b016] Thomson MJ, Tai TH, McClung AM, Lai XH, Hinga ME (2003). Mapping quantitative trait loci for yield, yield components and morphological traits in an advanced backcross population between Oryza rufipogon and the Oryza sativa cultivar Jefferson. Theor Appl Genet.

[pgen-0030133-b017] Jiang Y (2000). Role of anthocyanins, polyphenol oxidase and phenols in lychee pericarp browning. J Sci Food Agric.

[pgen-0030133-b018] Hagenblad J, Nordborg M (2002). Sequence variation and haplotype structure surrounding the flowering time locus FRI in Arabidopsis thaliana. Genetics.

[pgen-0030133-b019] Goff S, Cone K, Chandler V (1992). Functional analysis of the transcriptional activator encoded by the maize B gene: Evidence for a direct functional interaction between two classes of regulatory proteins. Genes Dev.

[pgen-0030133-b020] Nesi N, Clarisse J, Debeaujon I, Caboche M, Lepinec L (2001). The *Arabidopsis* TT2 gene encodes an R2R3 MYB domain protein that acts as a key determinant for proanthocyanidin accumulation in developng seed. Plant Cell.

[pgen-0030133-b021] Spelt C, Quattrocchio F, Mol J, Koes R (2002). ANTHOCYANIN1 of *Petunia* controls pigment synthesis, vacuolar pH, and seed coat development by genetically distinct mechanisms. Plant Cell.

[pgen-0030133-b022] Matsuo T, K Hoshikawa (1993). Science of the rice plant.

[pgen-0030133-b023] Harushimaa Y, Nakagahrab M, Yanoc M, Sasakic T, Kurata N (2002). Diverse variation of reproductive barriers in three intraspecific rice crosses. Genetics.

[pgen-0030133-b024] Messeguer J, Marfa V, Catala MM, Guiderdoni E, Mele E (2004). A field study of pollen-mediated gene flow from Mediterranean GM rice to conventional rice and the red rice weed. Mol Breed.

[pgen-0030133-b025] Li C, Zhou A, Sang T (2006). Rice domestication by reducing shattering. Science.

[pgen-0030133-b026] Konishi S, Izawa T, Lin SY, Ebana K, Fukuta Y (2006). An SNP caused loss of seed shattering during rice domestication. Science.

[pgen-0030133-b027] Yu J, Hu S, Wang J, Wong GK, Li S (2002). A draft sequence of the rice genome *(Oryza sativa* L. ssp. *indica)*. Science.

[pgen-0030133-b028] Goff SA, Ricke D, Lan TH, Presting G, Wang R (2002). A draft sequence of the rice genome *(Oryza sativa* L. ssp. *japonica)*. Science.

